# Coronary Calcium Scoring in Diabetes: Recalibrating Cardiovascular Risk in 2025

**DOI:** 10.1111/1753-0407.70178

**Published:** 2025-12-03

**Authors:** Kyvan Irannejad, Matthew Budoff

**Affiliations:** ^1^ Division of Cardiology Lundquist Institute Torrance California USA

**Keywords:** atherosclerosis, cardiovascular risk stratification, computed tomography, coronary artery calcium, diabetes mellitus, outcomes, prevention

## Abstract

CAC‐guided management framework for adults with diabetes.

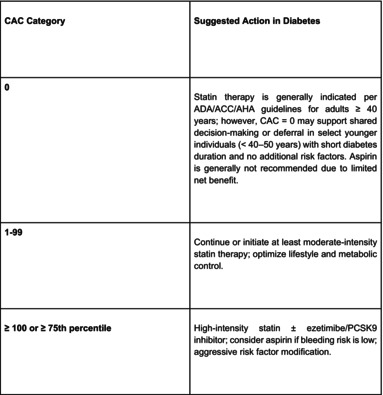

## 1 Introduction: Why Coronary Artery Calcium (CAC) is Transformative in Diabetic Care

Cardiovascular disease (CVD) remains the leading cause of death among individuals with diabetes mellitus (DM), accounting for nearly two‐thirds of mortality [1, 2]. Despite the arrival of glucose‐lowering therapies with proven cardiovascular benefit, including SGLT2 inhibitors and GLP‐1 receptor agonists, residual atherosclerotic risk persists [3, 4]. Traditional risk calculators, such as the pooled cohort equations and SCORE2‐Diabetes, treat diabetes as a categorical “risk equivalent,” assuming uniformly high atherosclerotic cardiovascular disease (ASCVD) risk across all individuals [5, 6]. Yet clinical experience and population data consistently refute this notion: many individuals with diabetes live decades without events, while others experience premature coronary artery disease despite optimal glycemic control.

In this context, CAC scoring, a non‐invasive, low‐radiation CT measure of calcified plaque burden, has redefined how clinicians visualize and quantify subclinical atherosclerosis [7, 8, 9]. The CAC score provides a *direct measure* of the cumulative exposure to metabolic and inflammatory injury, integrating lifetime risk in a single metric. In 2025, mounting evidence confirms that CAC scoring offers unmatched discrimination in predicting future CVD events among adults with diabetes, surpassing traditional risk factors, HbA1c, and even duration of diabetes [10, 11].

### 1.1 Landmark Cohort Studies

In the MESA cohort of 6751 adults, including 881 with diabetes, CAC proved a powerful predictor of cardiovascular events. Over 11 years of follow‐up, individuals with diabetes with CAC = 0 had very low CHD event rates (3.7 per 1000 person‐years), even with long disease duration or high calculated risk, while those with CAC ≥ 400 had rates exceeding 20 per 1000 person‐years. Traditional factors such as HbA1c, insulin use, or duration of diabetes did not add predictive value once CAC was included. Malik et al. concluded that CAC offers strong risk discrimination in diabetes; a zero score identifies low short‐term risk, whereas higher CAC scores mark a several‐fold increase in event rates [12]. A CAC score of zero identifies very low short‐term risk, whereas higher CAC scores mark a several‐fold increase in event rates. The predictive value of CAC is time‐dependent: in MESA, a zero score conferred a very low annual event rate (< 0.5%) for about 3–5 years in individuals with diabetes, after which risk gradually rose, whereas elevated CAC (≥ 100–400) predicted persistently higher event rates for a decade or more [13, 14].

Building on MESA, in a pooled analysis of 5174 adults (ages 38–55) from MESA and CARDIA, Masrouri et al. found that over 14 years of follow‐up, CAC, categorized as 0, 1–99, and ≥ 100, strongly predicted CVD, CHD, and mortality across metabolic groups. Overall, CVD incidence was 10.0, 6.7, and 3.1 per 1000 person‐years among participants with diabetes, metabolic syndrome, and neither condition, respectively. Among individuals with diabetes, those with CAC = 0 had event rates (~6.7 per 1000 person‐years) comparable to participants with metabolic syndrome but without diabetes, whereas CAC ≥ 100 conferred a two‐ to threefold higher risk of CVD, CHD, and mortality. In adjusted models, CAC ≥ 100 was associated with a 2.7‐fold higher CVD risk, 3.0‐fold higher CHD risk, and 3.7‐fold higher mortality in individuals with diabetes, with similar elevations in those with metabolic syndrome. The impact of CAC ≥ 100 was attenuated in diabetes (P for interaction < 0.02) yet remained significant after adjustment for HbA1c, underscoring CAC's role as an independent marker of atherosclerotic risk beyond glycemic control. Notably, HbA1c itself showed only a weak association with cardiovascular events once CAC was included, and no significant interaction was observed between HbA1c levels and CAC category [15].

In the DCCT/EDIC Type 1 Diabetes cohort, Budoff et al. evaluated 1205 participants followed for 10–13 years and found a strong graded association between CAC burden and incident CVD. Event rates increased from 5.6 to 37.9 per 1000 person‐years across CAC categories (0, > 0–100, > 100–300, > 300), corresponding to 5‐year absolute risks of 2.8%, 4.8%, 11.5%, and 17.3%. Compared with CAC = 0, adjusted hazard ratios for CVD were 4.05 for CAC > 100–300 and 4.73 for CAC > 300, and for major adverse cardiac events, 6.05 and 5.57, respectively (*p* < 0.0001). These findings indicate that risk rises sharply once CAC exceeds 100, with little incremental increase beyond 300, suggesting a plateau effect at higher scores. This pattern implies that in individuals with type 1 diabetes, a CAC threshold of > 100 may already capture those at maximal atherosclerotic risk, and that higher cutoffs confer only marginal additional prognostic value. Future studies with larger numbers of participants with very high CAC could further refine these upper thresholds [16].

### 1.2 Population‐Based and Regional Studies

Population‐based data extend these findings beyond well‐characterized academic research cohorts, demonstrating similar associations in community and regional populations. In Central Appalachia, Mamudu et al. screened 2479 asymptomatic adults and found subclinical atherosclerosis strikingly prevalent: 69% of individuals with diabetes had CAC > 0 versus 59% of those without diabetes. Mean CAC was 263 ± 563 in individuals with diabetes versus 136 ± 306 in controls. Among those with diabetes, hypertension, and physical inactivity increased the odds of CAC 100–399 by 3.8× and 2.5×, respectively. These data highlight geographic disparities and support CAC screening in high‐risk, underserved regions [17].

From the Harbor‐UCLA registry, Khazai et al. compared 861 individuals with diabetes with 861 matched controls. The presence of diabetes was strongly associated with both the presence and extent of CAC (*p* < 0.0001). Mean CAC was 343 versus 89; 84% of individuals with diabetes had CAC > 0, and mixed/calcified plaques were significantly more frequent (*p* = 0.018 and < 0.001, respectively), while non‐calcified plaque counts were similar (*p* = 0.40). Segment involvement, stenosis, and total plaque scores were all markedly higher, confirming more extensive and complex coronary disease [18].

### 1.3 CAC Progression and Therapy Response

Beyond baseline burden, progression of CAC adds independent prognostic value. In a seminal study of 596 asymptomatic adults, Sarkis et al. reported an average annual CAC increase of 29% ± 9% in individuals with diabetes and 10% ± 7% in controls (*p* < 0.0001). Mortality rose sharply with faster progression, HR 1.88 for ΔCAC 10%–20%, 2.29 for 21%–30%, and 6.95 for > 30% compared to < 10%. Statin therapy reduced all‐cause mortality by approximately 70% among individuals with diabetes who did not demonstrate CAC progression, whereas those with continued CAC progression derived less apparent benefit, underscoring that persistent plaque activity may signal residual risk despite lipid‐lowering therapy [19].

### 1.4 Cost‐Effectiveness and Implementation

Microsimulation modeling shows CAC‐guided prevention in adults with diabetes (40–75 years) is cost‐effective versus guideline‐based care, with base‐case incremental cost‐effectiveness ratios (ICERs) around $35 000–$50 000 per QALY, and < $25 000/QALY in sensitivity analyses (e.g., lower CAC test costs or improved adherence among non‐zero CAC groups). Savings arise from therapy intensification when CAC ≥ 100 and de‐intensification when CAC = 0, operationalizing CAC as a quantitative “risk thermometer” [20].

## 2 Controversies—The “CAC = 0 Paradox” in Diabetes

A persisting debate surrounds the so‐called “CAC = 0 paradox.” In the general population, a zero score conveys an annual cardiovascular event rate below 0.5%, representing a long “warranty period” of low risk. In individuals with diabetes, this warranty is notably shorter [13, 14]. In MESA, Malik et al. found that even with CAC = 0, individuals with diabetes experienced cardiovascular events over 10 years, though far fewer than those with CAC > 100 [12]. Kashif and Khazai at Harbor‐UCLA similarly observed that about 30% of individuals with diabetes, particularly younger women, maintained CAC = 0 for several years with low event rates (< 3 per 1000 person‐years). However, coronary CT angiography often reveals non‐calcified and mixed plaques despite a zero CAC score [18, 21]. Thus, the absence of calcification does not equate to the absence of atherosclerosis.

The pooled analysis by Masrouri et al. added nuance: CAC ≥ 100 predicted events across all metabolic groups, but hazard ratios were lower in diabetes (HR 2.7 vs. 6.3), suggesting a “ceiling effect” of metabolic injury. In individuals with diabetes, microvascular and non‐calcified disease may predominate, making CAC = 0 a marker of *lower*, not *absent*, risk. Long‐term cohort data indicate that the median time to CAC progression from 0 to > 0 is approximately 3–5 years in diabetes compared with 7–10 years in the general population, reflecting faster atherosclerotic evolution in diabetes [13, 14]. Therefore, repeating CAC scanning every 3–5 years is reasonable for individuals with diabetes, while 7–10 years remains appropriate for those without diabetes or major risk factors [13].

## 3 Clinical Implications—How Guidelines May Shift

### 3.1 Risk Stratification and Statin Allocation

Current ADA and ACC/AHA guidelines recommend at least moderate‐intensity statin therapy for all adults aged 40–75 years with diabetes, regardless of CAC status, with high‐intensity therapy favored in those with additional risk factors [22]. Emerging evidence, however, suggests that CAC scoring can refine the intensity and timing of therapy. Individuals with CAC = 0 represent a subgroup with particularly low short‐term risk, and CAC may inform a more personalized, discussion‐based approach, especially in younger adults (< 40–50 years) or those with short disease duration and no other major risk factors. In contrast, CAC ≥ 100 or ≥ 75th percentile consistently identifies individuals who benefit from aggressive lipid‐lowering and, in some cases, adjunctive therapies.

A summary of CAC‐guided therapeutic recommendations in adults with diabetes is provided in Table 1.

**TABLE 1:** CAC‐guided management framework for adults with diabetes.

CAC category	Suggested action in diabetes
0	Statin therapy is generally indicated per ADA/ACC/AHA guidelines for adults ≥ 40 years; however, CAC = 0 may support shared decision‐making or deferral in select younger individuals (< 40–50 years) with short diabetes duration and no additional risk factors. Aspirin is generally not recommended due to limited net benefit.
1–99	Continue or initiate at least moderate‐intensity statin therapy; optimize lifestyle and metabolic control.
≥ 100 or ≥ 75th percentile	High‐intensity statin ± ezetimibe/PCSK9 inhibitor; consider aspirin if bleeding risk is low; aggressive risk factor modification.

One study demonstrated that individuals with diabetes who did not exhibit CAC progression while receiving statin therapy had mortality rates comparable to those without diabetes, whereas individuals with CAC progression experienced the highest mortality [19]. Thus, CAC is both a risk identifier and a feedback biomarker for therapy success.

### 3.2 Beyond Statins—SGLT2i, GLP‐1RA, and Antiplatelet Decisions

While CAC scoring powerfully identifies individuals at elevated atherosclerotic risk, current evidence does not support using CAC thresholds alone to initiate cardioprotective glucose‐lowering therapies. The major outcome trials for SGLT2 inhibitors and GLP‐1 receptor agonists demonstrated cardiovascular benefit primarily among individuals with established ASCVD or multiple clinical risk factors, not those selected by imaging criteria [23, 24].

Nevertheless, a CAC ≥ 100 identifies adults with diabetes in whom absolute ASCVD risk approaches that seen in secondary‐prevention populations, suggesting that these individuals may derive meaningful relative and absolute benefit from agents with proven cardiovascular efficacy. Until prospective data are available, CAC should be regarded as a risk‐enhancing marker that can inform, but not independently determine, the decision to initiate an SGLT2 inhibitor or GLP‐1 receptor agonist, ideally within a shared decision‐making framework.

In primary prevention, CAC‐guided analyses suggest that aspirin offers a net clinical benefit when CAC ≥ 100 and bleeding risk is low, whereas individuals with CAC = 0 generally experience no net benefit, supporting avoidance of routine aspirin use in this group [22, 23, 24, 25].

### 3.3 Technological Advances

Artificial intelligence is transforming CAC assessment. Huangfu et al. developed AI‐derived *dispersion* and density metrics that improved the prediction of myocardial infarction and cardiovascular death, particularly in patients with diabetes, compared with traditional Agatston scoring [26, 27]. In parallel, fully automated “opportunistic” CAC quantification from routine, non‐gated chest CTs now allows identification of high‐risk patients without dedicated cardiac imaging [28, 29, 30]. With modern low‐dose CT protocols delivering < 1 mSv radiation and continuously falling costs, CAC screening is becoming as accessible as an HbA1c test, poised to enter routine preventive cardiometabolic care.

## 4 Conclusion—CAC As the Stethoscope of Preventive Cardiology

CAC scanning in diabetes has transitioned from a research tool to a cornerstone of precision prevention. It reveals the invisible vascular injury caused by chronic hyperglycemia, quantifies cumulative atherosclerotic burden, and personalizes therapy beyond traditional risk factors or HbA1c levels. Evidence from MESA, EDIC, and population studies shows that CAC reclassifies diabetes from a categorical “risk equivalent” to a spectrum of cardiovascular vulnerability, enabling tailored intensity of statin, antiplatelet, and cardiometabolic therapies. The “CAC = 0” paradox reminds clinicians that the absence of calcification signals lower, but not absent risk, warranting continued vigilance. As AI‐driven plaque analytics and opportunistic CAC detection become routine, clinicians can integrate imaging with metabolic data to guide individualized prevention. Ultimately, CAC has become the stethoscope of preventive cardiology in diabetes, allowing us to listen not to heart sounds, but to the silent whispers of atherosclerosis long before the first infarction.

## Funding

The authors have nothing to report.

## Conflicts of Interest

The authors declare no conflicts of interest.

## Data Availability

Data sharing not applicable to this article as no datasets were generated or analysed during the current study.
